# Notch1 signaling is limited in healthy mature kidneys in vivo

**DOI:** 10.1186/s13104-023-06326-x

**Published:** 2023-04-17

**Authors:** Ryosuke Sugiura, Takahiro Nakayama, Teppei Nishino, Naoto Sambe, Freddy Radtke, Masaharu Yoshihara, Satoru Takahashi

**Affiliations:** 1 College of Medicine, School of Medicine and Health Sciences, University of Tsukuba, 1-1-1 Tennodai, Tsukuba, Ibaraki, 305-8575 Japan; 2 Department of Anatomy and Embryology, Institute of Medicine, University of Tsukuba, 1-1-1 Tennodai, Tsukuba, Ibaraki, 305-8575 Japan; 3 Department of Medical Education and Training, Tsukuba Medical Center Hospital, 1-3-1 Amakubo, Tsukuba, Ibaraki, 305-8558 Japan; 4 School of Life Sciences, Ecole Polytechnique Fédérale de Lausanne (EPFL), Swiss Institute for Experimental Cancer Research (ISREC), SV 2534 (Bâtiment SV) Station 19, Lausanne, CH-1015 Switzerland; 5 PhD Program in Humanics, School of Integrative and Global Majors, University of Tsukuba, 1- 1-1 Tennodai, Tsukuba, Ibaraki, 305-8577 Japan; 6 Department of Primary Care and Medical Education, Institute of Medicine, University of Tsukuba, 1-1-1 Tennodai, Tsukuba, Ibaraki, 305-8575 Japan; 7 Laboratory Animal Resource Center in Transborder Medical Research Center, University of Tsukuba, 1-1-1 Tennodai, Tsukuba, Ibaraki, 305-8575 Japan

**Keywords:** Cre/loxP system, Delta-Notch signaling pathway, Disease model, Gal4/UAS system, Transgenic mouse

## Abstract

**Objective:**

A Delta-Notch signaling component, Notch1, is involved in the normal development and multiple disorders of the kidney. Although the increase in Notch1 signaling is crucial to these pathogeneses, the basal signaling level in ‘healthy’ mature kidneys is still unclear. To address this question, we used an artificial Notch1 receptor fused with Gal4/UAS components in addition to the Cre/loxP system and fluorescent proteins in mice. This transgenic reporter mouse system enabled labeling of past and ongoing Notch1 signaling with tdsRed or Cre recombinase, respectively.

**Results:**

We confirmed that our transgenic reporter mouse system mimicked the previously reported Notch1 signaling pattern. Using this successful system, we infrequently observed cells with ongoing Notch1 signaling only in Bowman’s capsule and tubules. We consider that Notch1 activation in several lines of disease model mice was pathologically significant itself.

**Supplementary Information:**

The online version contains supplementary material available at 10.1186/s13104-023-06326-x.

## Introduction

The Delta-Notch signaling pathway is a ligand‒receptor signaling pathway that mediates cell‒cell communication in developing organs [1]. Upon binding with Delta ligands, the Notch receptor intracellular domain is cleaved and acts as a transcription factor [2]. This signaling pathway is involved in multiple disorders as well as developmental processes. For example, Notch1 is reportedly involved in acute T-cell lymphoblastic leukemia [3], non-small cell lung carcinomas [4], and diabetic foot ulcerations [5]. In the kidney, Notch1 activation was observed in podocytes and tubulointerstitial cells from human patients with diabetic kidney disease (DKD) and focal segmental glomerular sclerosis (FSGS) [6]. Although the involvement of Notch1 in these disorders has been validated, its cellular mechanism is controversial. On the one hand, the Notch1 intracellular domain was expressed in podocytes of DKD and FSGS patients [7]. On the other hand, Notch1 was expressed in endothelial cells in the glomeruli, and overexpression of the Notch1 intracellular domain in Tie2-expressing endothelial cells led to albuminuria via decreased VE-cadherin expression [8]. To further characterize the involvement of Notch1 activity in these disorders, it is necessary to know the basal Notch1 activity in healthy subjects. A previous study suggested that Notch1 activity in the ‘healthy’ mature kidney was very low using a transgenic mouse line (N1IP::Cre(LO), formerly known as NIP-CRE) that expressed Notch1-Cre fusion protein [9]. The same group, however, generated the second‒generation Notch1-Cre mouse (N1IP::CreHI), which improved the detection sensitivity of Notch1 activity to demonstrate past Notch1 signaling in epithelial cells from the proximal to distal tubule of the mature kidney [10]. Indeed, single-cell RNA-seq revealed that Notch1 signaling promoted the maturation of all nephron segments and selection of proximal tubular cell fate [11]. In contrast to its developmental contribution, ongoing Notch1 signaling in the ‘healthy’ mature kidney is still unclear.

**Fig. 1:**
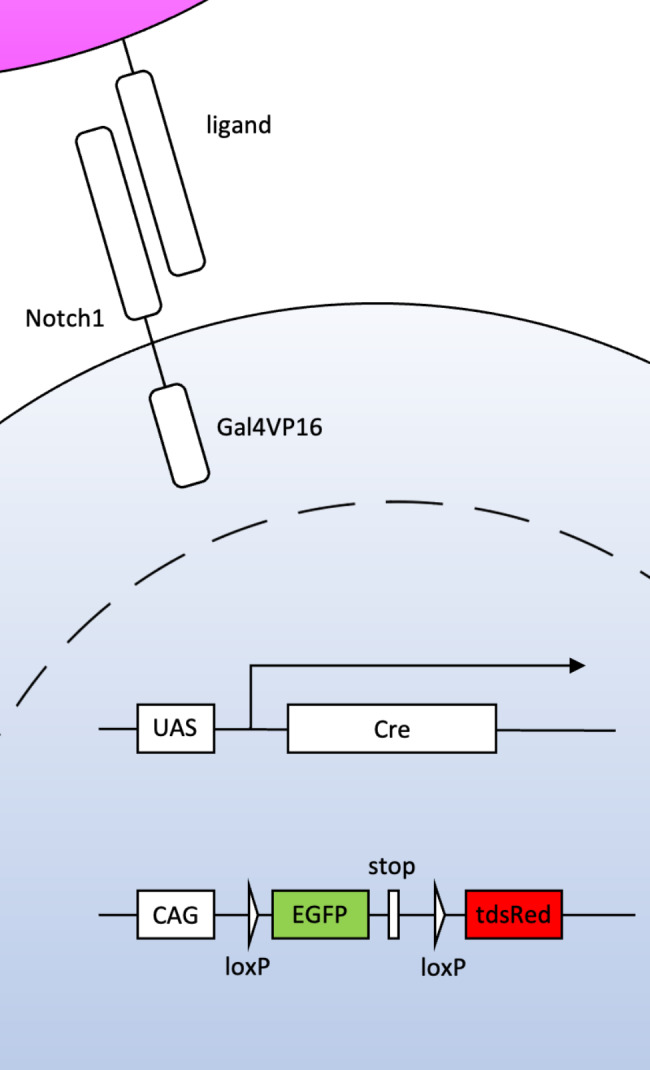
Strategy for visualization of past and ongoing Notch1 signaling By combining three transgenic mouse lines, Notch1 binding to its ligands induces the expression of Cre recombinase (ongoing Notch1 signal), which eventually results in tdsRed expression (past Notch1 signal).

Here, we examined ongoing Notch1 signaling in mature kidneys using our original reporter mouse system that consisted of three transgenic mouse lines. The first mouse line expresses the artificial Notch1 receptor, the intracellular domain of which was replaced with the yeast transcription factor Gal4VP16 (N1-Gal4VP16 mouse) [12]. The second transgenic mouse line expresses Cre recombinase when Gal4VP16 binds to its upstream activating sequence (UAS) (UAS-Cre mouse) [13]. These two mouse lines enable visualization of ongoing Notch1 signaling by immunohistochemistry for Cre recombinase. The third mouse line expresses EGFP or tandem dsRed (tdsRed) before and after Cre-mediated recombination, respectively. In detail, the tdsRed coding sequence was inserted into the *ROSA26* locus following a floxed EGFP-STOP sequence (R26GRR mouse) [14]. Therefore, past and ongoing Notch1 signals are labeled with tdsRed or Cre recombinase, respectively (Fig. 1).

TdsRed protein was detected in proximal tubular epithelial cells, suggesting that our reporter mouse system worked. Using this working system, we examined ongoing Notch1 signaling in the ‘healthy’ mature kidney.

## Materials and methods

### Animal Welfare

Animal experiments were carried out in accordance with the Regulation for Animal Experiments in our university and Fundamental Guidelines for Proper Conduct of Animal Experiment and Related Activities in Academic Research Institutions under the jurisdiction of the Ministry of Education, Culture, Sports, Science and Technology. Approval was obtained from the Institutional Animal Care and Use Committee and the DNA Experiment Committee of the University of Tsukuba (Approval Numbers for Animal Experiments: 22–059) (Approval Number for DNA Experiments: 220018). UAS-Cre mice are available from RIKEN through the National BioResource Project of Japan: Crl:CD1(ICR)-Tg(UAS-cre/T2A/miRFP670)216Staka (No. RBRC11716). In addition, R26GRR mice are also available from RIKEN through the National BioResource Project of Japan: C57BL/6 N-Gt(ROSA)26Sor < tm1(CAG-EGFP/tDsRed)Utr>/Rbrc (No. RBRC04874).

### Histological analysis

We used three N1-Gal4VP16; UAS-Cre; R26GRR mice (10–13 weeks old), one UAS-Cre; R26GRR mouse (6 weeks old) and one Non-Tg mouse (5 weeks old) for the analysis. To reduce the number of mice used, we used post-weaning single mouse per group for the negative control (UAS-Cre; R26GRR and Non-Tg groups). Prior to sampling the organs, mice were sacrificed by cervical dislocation and perfused with PBS and Mildform 10 N (Cat #: 133-10311, Fujifilm, Osaka, Japan). For fluorescent imaging, 10-µm-thick frozen sections were counterstained with Hoechst 33342 (Cat #: H3570, Invitrogen, Waltham, MA, USA). For immunohistochemistry, 4-µm-thick paraffin sections were sequentially incubated with a rabbit monoclonal anti-Cre recombinase antibody (clone: D7L7L, Cat #: 15036 S, RRID: AB_2798694, Cell Signaling Technology) or a rabbit anti-RFP antibody (cat #: 600-401-379, Lot #: 46317, RRID: AB_2209751, Rockland Immunochemicals, Philadelphia, PA, USA), Histofine SimpleStain (Cat #: 414341, Nichirei Biosciences, Tokyo, Japan), Histofine DAB Substrate Kit (Cat #: 425011, Nichirei Biosciences) and counterstained with Mayer’s hematoxylin solution (Cat #: 131–09665, Fujifilm, Osaka, Japan). Images were captured by using BIOREVO-BZ-X810 (Keyence, Osaka, Japan).

## Results and discussion

### Examination of past notch1 signaling in ‘healthy’ mature kidneys

**Fig. 2:**
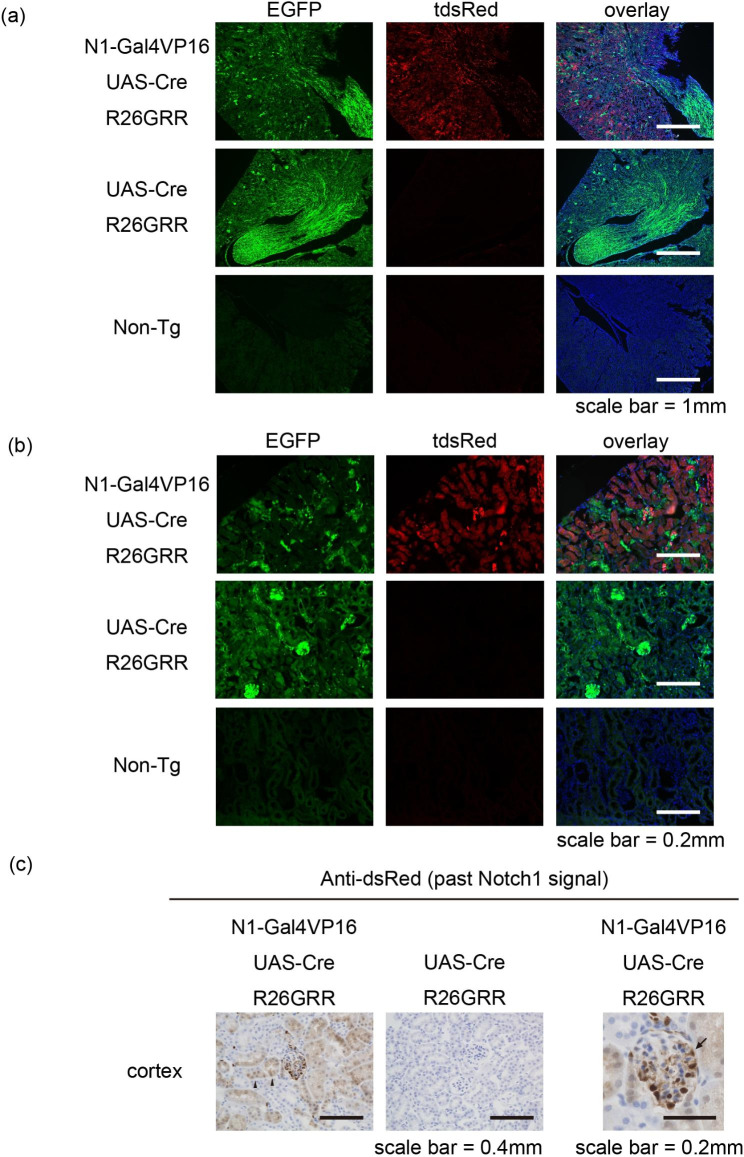
Visualization of past Notch1 signaling in ‘healthy’ mature kidneys (a) Fluorescent examination of ‘healthy’ mature kidney at a low magnification. Note that tdsRed was expressed in both the cortex and medulla specifically in N1-Gal4VP16; UAS-Cre; R26GRR mice. (b) Fluorescent examination of ‘healthy’ mature kidney at a high magnification. Note that tdsRed was expressed in the tubules. (c) Immunohistochemistry for dsRed showed that proximal tubular epithelial cells (arrowheads) and podocytes (arrow) had a Notch1 signal in concordance with previous reports.

To examine whether our reporter mouse system works, we started by visualizing the past Notch1 signal (tdsRed expression) in the ‘healthy’ mature kidney. Fluorescent imaging showed tdsRed expression in the cortex and medulla specifically in N1-Gal4VP16; UAS-Cre; R26GRR mice (Fig. 2a). We observed tdsRed fluorescence in the tubular epithelium and in the glomeruli (Fig. 2b). Furthermore, immunohistochemistry for dsRed protein showed that most of the proximal tubular epithelial cells and podocytes were positive, in concordance with previous reports [10, 11], suggesting that our reporter mouse system worked (Fig. 2c).

### Examination of ongoing notch1 signaling in ‘healthy’ mature kidneys

**Fig. 3:**
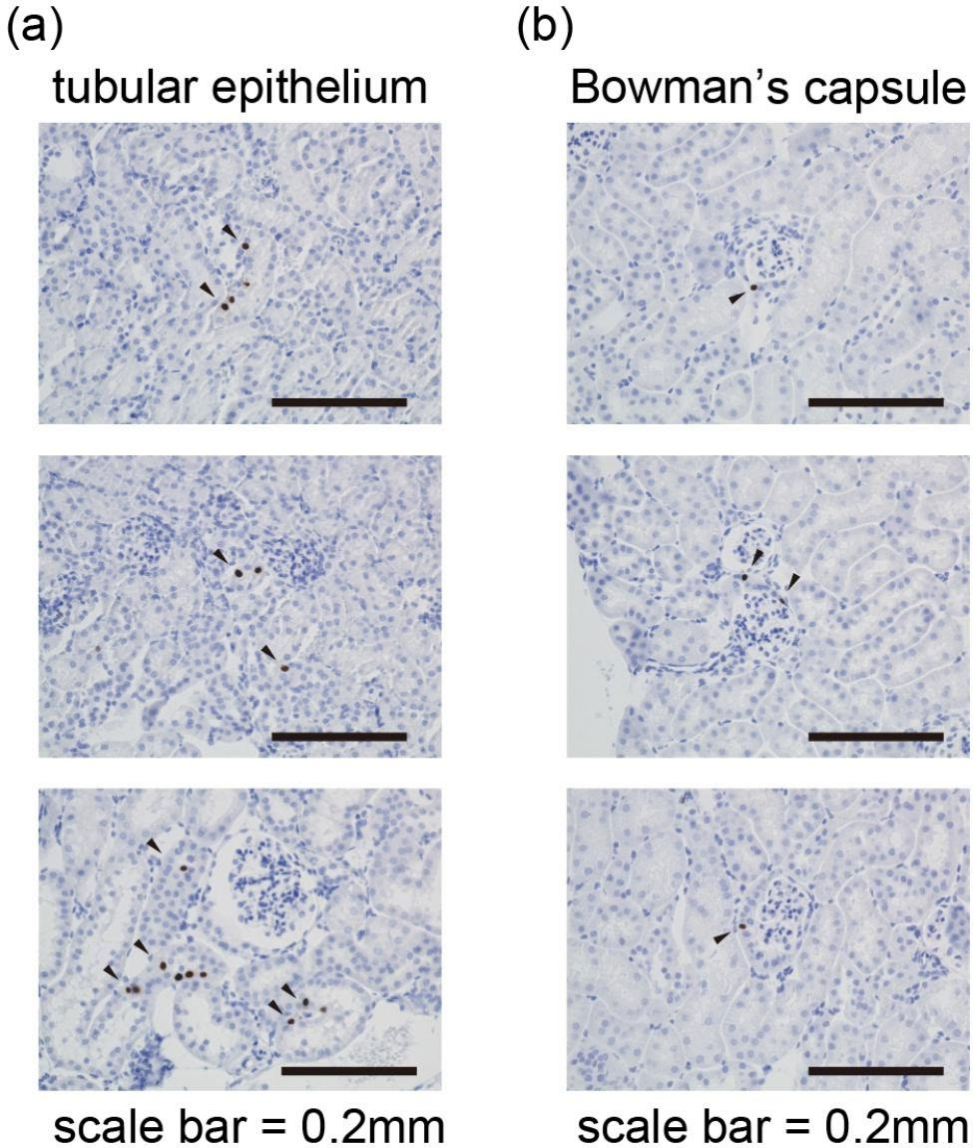
Representative images of Cre-expressing cells in ‘healthy’ mature kidneys Immunohistochemistry for Cre recombinase showed occasional positive cells in the tubular epithelium (a) and Bowman’s capsule (b).

Next, we examined ongoing Notch1 signaling using our reporter mouse system. Overall, Cre-expressing cells were very rare (less than 1% of total kidney cells). Indeed, we never observed Cre-expressing cells in the medulla. In contrast, we occasionally observed these cells in the tubular epithelium (Fig. 3a) and Bowman’s capsule (Fig. 3b). As such, Bowman’s capsule has a pillar or cuboidal epithelium, and Notch1 signaling might be induced following activation of Bowman’s capsule. Collectively, we consider that Notch1 activation in several lines of disease model mice [6–8] was pathologically significant itself.

Finally, to examine our finding that Notch1 signaling in ‘healthy’ mature kidney is rare, we conducted a single-cell RNA-sequencing analysis using a previously reported dataset (GSE107585) [15]. The methods and results were described in “Additional file 1”, and the source code used to generate the results is available at GitHub repository (https://github.com/MasaharuYoshihara/Notch1-Signal-Kidney).

## Conclusion

We confirmed that proximal tubular epithelial cells underwent Notch1 signaling. In contrast, cells with ongoing Notch1 signaling were very rare in ‘healthy’ mature kidneys.

### Limitations

We were unable to describe the specific characteristics of Cre-expressing cells, although those cells tended to be located near each other. That was one reason the present study lacks explanation on the mechanism behind ongoing Notch1 signaling in the ‘healthy’ mature kidney.

## Electronic supplementary material

Below is the link to the electronic supplementary material.

Supplementary Material 1: Additional file 1: Single-cell RNA-sequencing analysis of healthy mature mouse kidney.

## Data Availability

The datasets generated and/or analyzed during the current study are available in the figshare repository, 10.6084/m9.figshare.21331212.v1.

## Abbreviations

DKD: diabetic kidney disease
FSGS: focal segmental glomerular sclerosis
tdsRed: tandem dsRed
UAS: upstream activating sequence
