# Supplementary material for: Notch1 signaling is limited in healthy mature kidneys in vivo
Source: BMC Res Notes. 2023 Apr 17;16:54. doi: 10.1186/s13104-023-06326-x (PMC10111784; doi:10.1186/s13104-023-06326-x)
Supplement: Supplementary file 1 — Supplementary Material 1: Additional file 1: Single-cell RNA-sequencing analysis of healthy mature mouse kidney. [file 13104_2023_6326_MOESM1_ESM.pdf]

Additional file 1

single-cell RNA-sequencing analysis of healthy mature mouse kidney

This file contains the methods, results, and discussion of single-cell RNA-sequencing analysis of healthy mature mouse kidney. We downloaded processed data (GSE107585) (1) from Gene Expression Omnibus (https://www.ncbi.nlm.nih.gov/geo/). The downloaded data, containing 43745 cells and 16273 gene symbols, were subjected for quality control using Scanpy (version 1.7.2) (2). We used 43310 cells and 1296 gene symbols for final analysis (See "Sugiura-ver1.ipynb" for quality control). Single-cell RNA-sequencing analysis was completed in Python 3.6.13 in an Ubuntu 20.04 LTS environment.

We ran principal component analysis followed by uniform manifold approximation and projection (UMAP), and identified 12 clusters that was annotated by the gene markers used in the original report (1) (Supplementary Figures 1a and 1b). To clearly illustrate the marker gene expressions, we standardized the dimension between 0 and 1 for each variable for Supplementary Figure 1b. Then, we examined the expression levels of Notch receptors (Notch1,2,3,4) and showed that Notch1 and Notch4 shared enrichment in cluster #7 (endothelial cells). Notch2 was expressed in cluster #8 (macrophages) and #11 (collecting duct intercalated cells) whereas Notch3 was rarely expressed (Supplementary Figure 1c).

Next, we examined Notch downstream target genes (Hes/Hey/Nrarp) (Supplementary Figure 1d). Hes3, Hes4 and Hes5 were not considered as differentially expressed genes. Among other Hes/Hey/Nrarp genes, Hes1 was expressed in cluster #7 (endothelial cells) along with clusters #3 (collecting duct transitional cell) and #11 (collecting duct intercalated cells). Importantly, the expression levels of Notch1, Notch4 and Hes1 in cluster #7 (endothelial cells) were relatively low. Note that we did not standardize the dimension between 0 and 1 for each variable for Supplementary Figures 1c and 1d to illustrate the unbiased expression levels. Along with these low expression levels, since both Notch1 and Notch4 were expressed in cluster #7 (endothelial cells), it remained uncertain that Notch1 signaling mediated Hes1 expression in this cell population. Collectively, we believe that our experimental finding that Notch1 signaling is rare is reasonable.

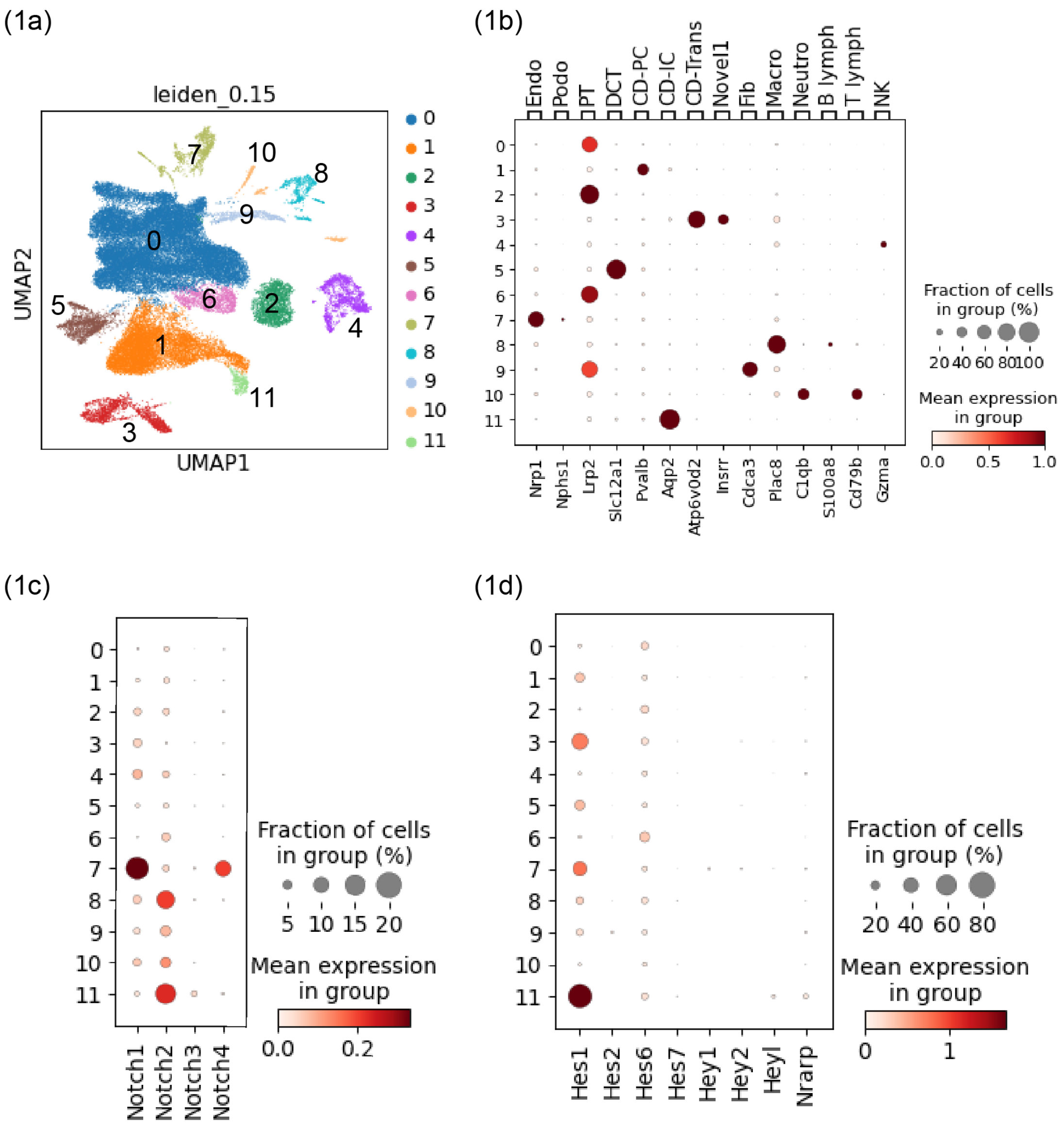

(1a)
leiden_0.15
UMAP1
UMAP2
0 1 2 3 4 5 6 7 8 9 10 11
(1b)
Endo Podo PT DCT CD-PC CD-IC CD-Trans Novel1 Fib Macro Neutro B lymph T lymph NK
Nrp1 Nphs1 Lrp2 Slc12a1 Pvalb Aqp2 Atp6v0d2 Insrr Cdca3 Plac8 C1qb S100a8 Cd79b Gzma
Fraction of cells in group (%)
20 40 60 80 100
Mean expression in group
0.0 0.5 1.0
(1c)
Notch1 Notch2 Notch3 Notch4
Fraction of cells in group (%)
5 10 15 20
Mean expression in group
0.0 0.2
(1d)
Hes1 Hes2 Hes6 Hes7 Hey1 Hey2 Heyl Nrarp
Fraction of cells in group (%)
20 40 60 80
Mean expression in group
0 1

Supplementary figure legends

(1a) Twelve clusters were identifiable in UMAP. (1b) Dot plots showing annotation of each cluster. See Abbreviations section below for the meaning of each cluster name. (1c) Dot plot of Notch receptor expressions. Note that Notch1 and Notch4 were expressed in cluster #7 (endothelial cells). (1d) Dot plot of Notch downstream target gene (Hes/Hey/Nrarp) expressions. Note that Hes1 were expressed in cluster #3 (collecting duct transitional cell), #7 (endothelial cells) and #11 (collecting duct intercalated cells).

Abbreviations

Podo, podocyte; PT, proximal tubule; LOH, ascending loop of Henle; DCT, distal convoluted tubule; CD-PC, collecting duct principal cell; CD-IC, collecting duct intercalated cell; CD-Trans, collecting duct transitional cell; Fib, fibroblast; Macro, macrophage; Neutro, neutrophil; lymph, lymphocyte; NK, natural killer cell; UMAP, uniform manifold approximation and projection.

Reference

1. Park J, Shrestha R, Qiu C, Kondo A, Huang S, Werth M et al. Single-cell transcriptomics of the mouse kidney reveals potential cellular targets of kidney disease. Science.

2018;360(6390):758-763.

2. Wolf FA, Angerer P, Theis FJ. SCANPY: large-scale single-cell gene expression data analysis. Genome Biol. 2018;19(1):15.
